# Supplementary material for: A whole-animal phenotypic drug screen identifies suppressors of atherogenic lipoproteins
Source: eLife. 2026 Aug 27;14:RP105314. doi: 10.7554/eLife.105314 (PMC13521566; doi:10.7554/eLife.105314)
Supplement: Supplementary file 2 [file elife-105314-code1.zip › JHDL_primary_result/Doxycycline/Doxycycline_JHDL_17_003_043_2019_09_13_12_21.pdf]

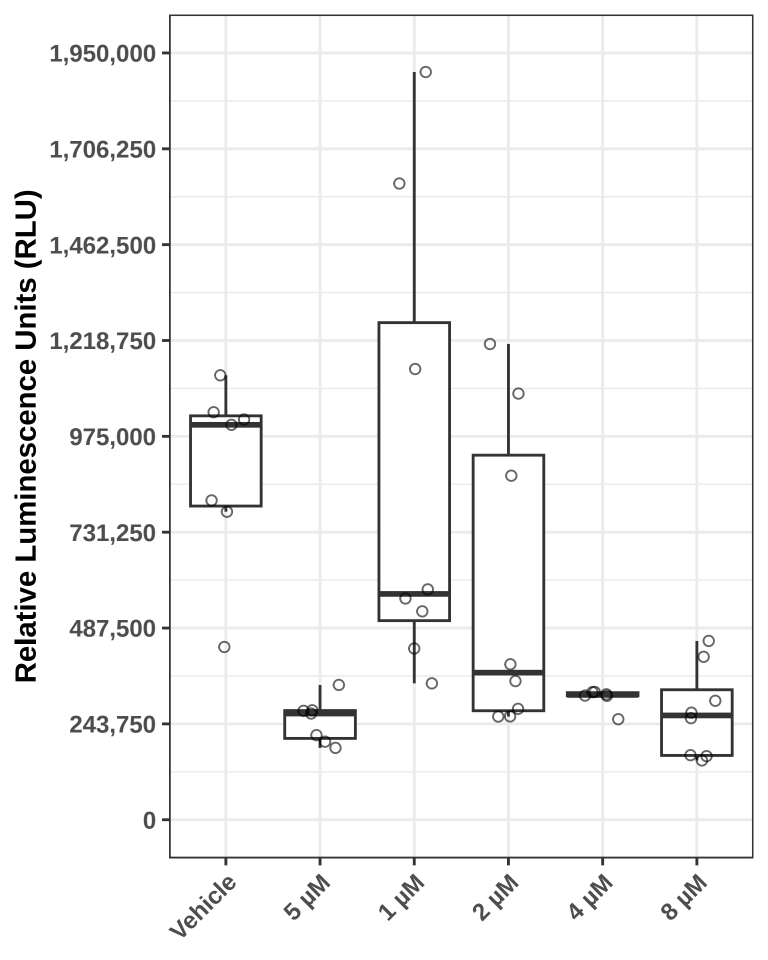

Relative Luminescence Units (RLU)
1,950,000
1,706,250
1,462,500
1,218,750
975,000
731,250
487,500
243,750
0
Vehicle
5 µM
1 µM
2 µM
4 µM
8 µM
